# Growth hormone-releasing hormone signaling and manifestations within the cardiovascular system

**DOI:** 10.1007/s11154-024-09939-0

**Published:** 2025-01-30

**Authors:** Raul A. Dulce, Konstantinos E. Hatzistergos, Rosemeire M. Kanashiro-Takeuchi, Lauro M. Takeuchi, Wayne Balkan, Joshua M. Hare

**Affiliations:** 1 Interdisciplinary Stem Cell Institute, University of Miami Miller School of Medicine, Biomedical Research Building, 1501 N.W. 10th Avenue, Room 908, Miami, FL 33136 USA; 2 Department of Genetics, Development and Molecular Biology, School of Biology, Aristotle University of Thessaloniki, Thessaloniki, 54124 Greece; 3 Department of Molecular and Cellular Pharmacology, University of Miami Miller School of Medicine, Miami, FL 33136 USA; 4 Department of Medicine, University of Miami Miller School of Medicine, Miami, FL 33136 USA

**Keywords:** Growth hormone-releasing hormone, GHRH-analogs, Cardiovascular, Cardioprotective, GHRH receptors

## Abstract

Growth hormone (GH)-releasing hormone (GHRH), a hypothalamic peptide initially characterized for its role in GH regulation, has gained increasing attention due to its GH-independent action on peripheral physiology, including that of the cardiovascular system. While its effects on the peripheral vasculature are still under investigation, GHRH and synthetic agonists have exhibited remarkable receptor-mediated cardioprotective properties in preclinical models. GHRH and its analogs enhance myocardial function by improving contractility, reducing oxidative stress, inflammation, and offsetting pathological remodeling. Studies performed in small and large animal models have demonstrated the efficacy of these compounds in diverse cardiomyopathies, suggesting their potential as promising therapeutic agents. However, the clinical translation of GHRH synthetic analogs still faces challenges related to the route of administration and potential side effects mainly associated with activation of the GH/IGF-I axis. Despite these hurdles, the compelling evidence supporting their role in cardiac repair makes GHRH analogs attractive candidates for clinical testing in the treatment of various cardiac diseases.

## Introduction

Growth hormone-releasing hormone (GHRH) is a hypothalamic peptide that stimulates the release of growth hormone (GH) from the anterior pituitary gland [1]. Human hypothalamic GHRH (also denominated growth hormone-releasing factor (GHRF), somatoliberin and somatorelin), is a 44 amino acid peptide [2], originally characterized from human pancreatic tumors [3, 4]. Its GH-releasing activity resides in the first 29 N-terminal amino acids [5, 6]. While primarily associated with growth and development, GHRH has also been implicated in various physiological processes, including cardiovascular function. Thus, the expression and function of the GHRH receptor (GHRHR, a G protein-coupled receptor) signaling extends beyond the hypothalamic-pituitary axis [7–9]. In the 1980’s, Hasegawa and colleagues [10] demonstrated that GHRH exerted positive inotropic effects on isolated papillary muscle, providing an early indication of GHRH acting directly on the myocardium. In recent years, there has been growing interest in the potential therapeutic applications of synthetic analogs of GHRH for a variety of diseases. Manipulation of the N-terminal amino acid sequence yielded a series of peptides that possess considerably enhanced potency in vitro and in vivo in diverse animal species [11, 12]. This development of new potent GHRH analogs and their application in different models of ischemic [11, 13–17] or non-ischemic [18–21] myocardial injury demonstrated that activation of myocardial GHRHRs effectively improve several aspects of cardiac function and hemodynamics as well as activation of survival and reparative pathways [13–16, 19, 21, 22].

Here we present a comprehensive overview of the direct effects of GHRH and synthetic analogs on the cardiovascular system, with particular emphasis on the actions of these analogs on the myocardium, highlighting their mechanisms of action and potential clinical applications.

## The role of GHRH/GHRHR signaling in development and growth

The development of animal models with GHRH or GHRHR gene knockouts or functional inactivation yielded valuable insights into the roles of GHRH/GHRHR during both developmental and postnatal stages. In 1976, a spontaneous occurrence of dwarfism was observed in a C57Bl/6J mouse colony at Jackson Laboratory, resulting in one dwarf female and two dwarf male offspring. These mice exhibited growth hormone and prolactin deficiencies, mirroring the human isolated growth hormone deficiency type I (IGHD) phenotype. Genetic analysis revealed a novel autosomal recessive mutation, termed “*little*” (*lit*^*−/−*^), on chromosome 6 [23]. Further research identified that the *lit* mutation was a missense mutation in the extracellular domain of the GHRHR protein, specifically a single nucleotide substitution that replaced aspartic acid with glycine at position 60 of GHRHR. These findings underscored the critical role of GHRHR in regulating postnatal linear growth [23, 24]. Similarly, GHRH knockout mice display reduced GH/IGF-1 secretion and exhibit dwarfism, closely resembling the phenotypes observed in *lit*^*–/–*^ mice and IGHD [25, 26].

Further phenotypic studies of *lit*^*–/–*^ [27] and GHRH knockout mice [25, 26] suggest that their growth retardation may be linked to a circadian rhythm-dependent metabolic mechanism, with GHRH playing a regulatory role primarily during the light but not the dark cycle. Specifically, disruption of GHRH/GHRHR signaling leads to increased fatty acid utilization, lowered plasma glucose levels, and heightened insulin sensitivity. These metabolic shifts result in reduced energy expenditure, decreased lean body mass, and increased fat accumulation [25–27]. These observations align with recent findings that hypothalamic GHRH^+^ neurons act as critical glucose sensors within the central nervous system, becoming activated in response to hypoglycemia [28], highlighting the crucial role of GHRH/GHRHR signaling in glucose metabolism. Interestingly, glucose-sensing neurons also have significant effects on cardiovascular metabolism [28]. Noteworthy, the GHRH analog tesamorelin has been FDA-approved for the treatment of HIV-related lipodystrophy [29, 30].

**Fig. 1:**
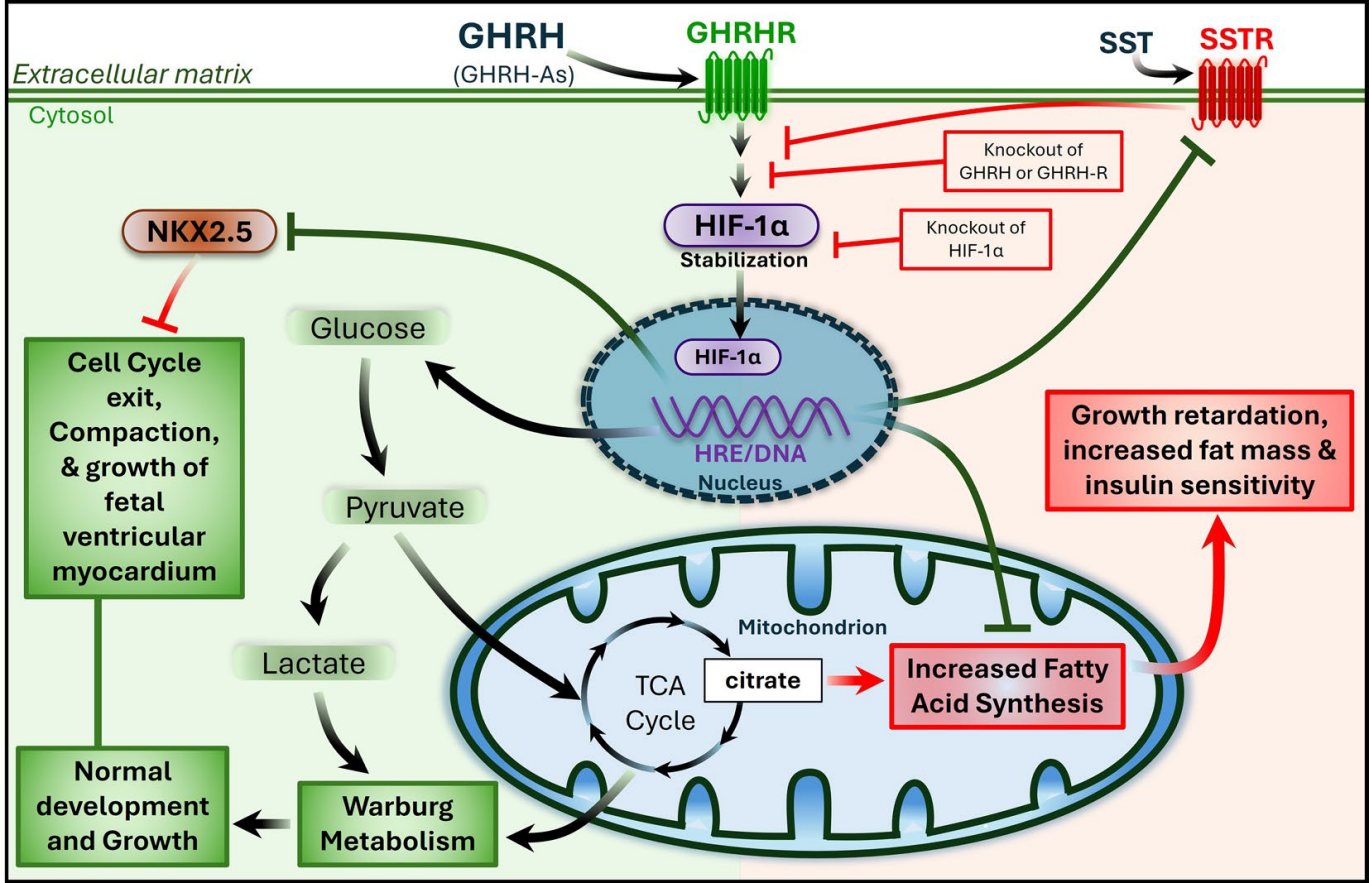
Proposed Role of GHRH/GHRHR in the Metabolic Control of Heart Development. During development, cardiac cells switch between glucose and fatty acid metabolism. In NKX2.5 + cardiac progenitor cells, autocrine activation of GHRH/GHRHR stimulates oxygen-independent, cell-specific, localized activation of HIF-1α. This activation represses NKX2.5 expression, promoting a shift to aerobic glycolysis (Warburg metabolism), cell cycle exit, and thickening growth of the ventricular wall (compaction). Conversely, inactivation of GHRH/GHRHR (or HIF-1α) maintains NKX2-5 expression, redirecting glycolytic products toward fatty acid synthesis, which supports cardiac cell cycle reentry and trabecular cardiomyogenesis, at the expense of ventricular compaction. SST, somatostatin; SSTR, SST receptor; TCA, tricarboxylic acid; Adapted from *Wanschel ACBA et al. 2023*,* BioRxiv 2023:2022.2001.2031.478572* [33].

The precise role of GHRH/GHRHR signaling in embryonic growth and development versus postnatal growth and homeostasis remains unclear. Initial studies on *lit*^*−/−*^ mice observed that mutant newborns tended to have lower birth weights, suggesting that the effects of disrupted GHRH/GHRHR signaling might already be present at birth [23]. Additionally, GHRH signaling increases the activity of tyrosine hydroxylase (TH)^+^ neurons in chick embryos independently of growth hormone, indicating that GHRH plays important roles in neurogenesis [31]. In mice, GHRH^+^/TH^+^ neurons in the hypothalamus originate from a neuronal lineage expressing the homeobox transcription factors *Isl1* and *Nkx2-1*. Disruption of *Isl1* in the *Nkx2-1* lineage leads to the complete loss of hypothalamic GHRH^+^ neurons, impaired glucose metabolism, and growth retardation [32]. Similarly, recent research from our group has identified both GHRH and GHRHR expression during development in human myocardial progenitors and cardiomyocytes that express *Isl1* and *Nkx2-5* [33]. Interestingly, GHRH/GHRHR signaling in cardiac progenitors appears to be regulated autonomously, independent of GH/IGF-1 activation and hypothalamic GHRH expression [33]. Consistent with the glucose-sensing role of GHRH in neurons [28] and the enhanced insulin sensitivity observed in GHRH/GHRHR-deficient mice [25–27], GHRH/GHRHR activation during cardiomyogenesis enhances glycolysis by promoting oxygen-independent stabilization of the transcription factor HIF-1α. In contrast, genetic inactivation of HIF-1α diminishes the effects of GHRH/GHRHR and shifts glucose metabolism in cardiomyocytes toward long-chain fatty acid synthesis [33] (Fig. 1). This HIF-1α-dependent effect of GHRH/GHRHR in preventing glucose conversion into fatty acids may also help explain the increased fat mass and decreased lean mass seen in GHRH/GHRH-R-deficient mice [25–27].

## GHRH on the peripheral vascular system

There is limited experimental evidence that GHRH exerts vasodilatory effects, whereas there is abundant literature supporting blood pressure reduction mediated by activation of the GH/insulin-like growth factor-I (IGF-I) axis in vivo [34, 35]. Despite the expression of GHRHRs in endothelial cells [36], a direct coronary or peripheral vasodilatory action of GHRH (or synthetic analogs) has not been properly demonstrated. GHRH-induced nitric oxide (NO) is produced in somatotroph cells [37] to potentiate GH production, and generation of cGMP occurs in cardiomyocytes upon stimulation with GHRH and synthetic analogs [18] (indicating activation of the soluble guanylyl cyclase (sGC)/cGMP/protein kinase C (PKC) pathway, usually mediated by activation of sGC by NO). However, it remains uncertain whether GHRH or GHRH analogs can trigger the activation of endothelial nitric oxide synthase (eNOS/NOS3) to promote vasodilation. In contrast, there is robust evidence demonstrating that GHRH and its analogs have substantial effects on the structure of the vascular system [38]. The angiogenic potential of either mesenchymal stem cells or endothelial progenitor stem cells was enhanced by GHRH and synthetic agonists both in vitro and in vivo [39–41], an effect capable of attenuating the vascular resistance. In this regard, GHRH agonists also induced an increase in myocardial capillary density in models of heart failure (HF) [14, 17, 20]. Antagonists of the GHRHR have anti-angiogenic properties in tumors by inhibiting VEGF secretion and endothelial cell proliferation [42], which supports the angiogenic role of GHRH. In this regard, GHRH in tumors is associated with enhanced expression of inducible NOS (iNOS/NOS2), which mediates pathogenic oxidative and nitrosative stress [43]. Interestingly, GHRH plays a role in regulating vascular calcification of the aorta and heart valves by inhibiting osteogenesis [44, 45]. These effects are mediated by antioxidant and anti-inflammatory activity, inhibition of the osteogenic transcription factor Runx2 expression and lowering of alkaline phosphatase levels. In the study by Ren et al. using diabetic mice, the GHRH synthetic agonist MR-409 improved the endothelium-dependent relaxation and reduced vascular injury without changes in the plasma level of GH [45]. However, a robust body of preclinical studies shows that GHRH antagonists protect against endothelial barrier dysfunction [46], probably by favoring the unfolded protein response (UPR) mechanisms, leading to activation of p53 [47] and, thus, counteracting redox imbalance, endothelial inflammation [36, 48–54] and consequent vascular remodeling. This information suggests that GHRHR-signaling might have deleterious effects on endothelial permeability. The GHRH antagonist MIA-602, demonstrated to be protective in a SARS-CoV-2 infection mouse model, attenuated the cardiopulmonary injury and lung perivascular inflammation [55]. In vitro experiments with peripheral blood mononuclear cells (PBMCs) stimulated with SARS-CoV-2 spike protein and LPS demonstrated that MIA-602 exerts antioxidant and anti-inflammatory effects [56], supporting the findings from Condor Capcha et al. [55]. Accordingly, an important factor with relevance to endothelial function is the role of GHRH on the circulatory component of the immune system [57]. GHRHRs are expressed and GHRH is secreted from PBMCs [58, 59], and although pro-inflammatory pathways are activated by GHRH in these cells [43, 60], this hormone is required for a healthy and better constitution of the immune cellular profile [61, 62]. The modulatory role of GHRH on this profile and its activity is highly dependent on the age, sex and neuroendocrinological regulation of each individual [57].

## GHRH on myocardial function and structure

**Fig. 2:**
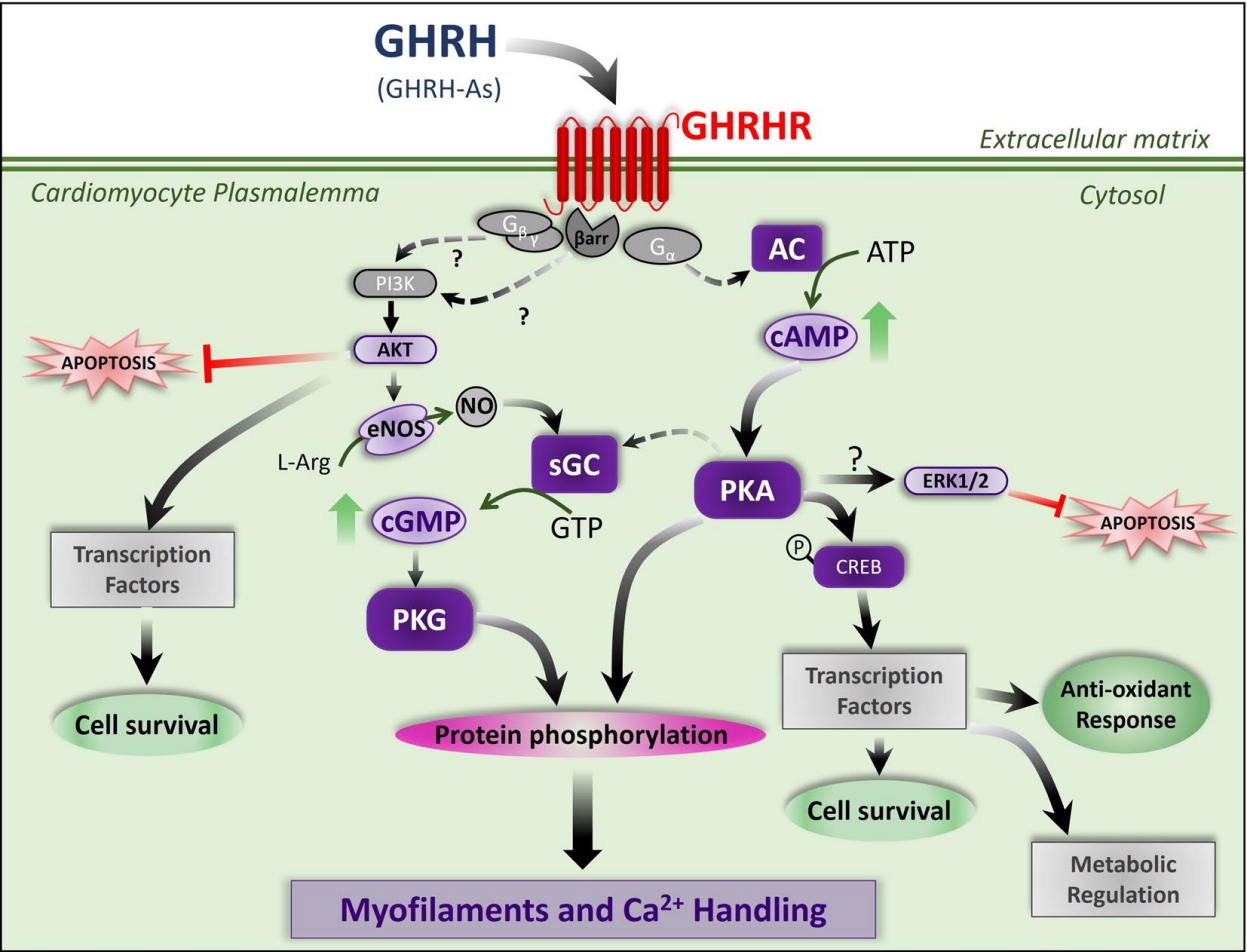
Proposed mechanism of action for GHRH agonists on the modulation of calcium signaling, contractility and reparative mechanisms in myocardial cells. Activation of GHRHRs (GPCR) induces the adenylyl cyclase (AC)/cyclic AMP/protein kinase A (PKA) pathway. PKA can both phosphorylate sarcomeric and calcium handling proteins and activate soluble guanylyl cyclase (sGC). This pathway also promotes antioxidant, reparative and survival mechanisms at transcriptional level. GHRHRs signaling also promotes activation of AKT (PKB) and ERK1/2 mediate the anti-apoptotic effects of GHRH agonists (GHRH-As). Endothelial Nitric Oxide Synthase (eNOS) is proposed to be activated by GHRH. The generated nitric oxide (NO) in turn activates sGC to produce the second messenger cGMP, an activator of protein kinase G (PKG). This kinase is also able to modulate myofilament and calcium cycling. This pathway has been proposed to be activate downstream GHRHRs through activation of PI3K by the Gβγ. proteins or β-arrestins (among other possible mechanisms). Adapted from *Dulce RA et al. 2022*,* Cardiovasc Res* [18].

GHRH and synthetic analogs exert direct effects on the myocardium, beyond their GH-releasing activity, including a broad spectrum of cardiac physiological effects, such as augmenting cardiac contractility by improving sarcoplasmic reticulum (SR) function, enhancing calcium handling in cardiomyocytes as well as targeting myofilaments. These effects may be mediated through the activation of myocardial GHRHRs and downstream signaling pathways. GHRH and GHRH synthetic analogs prevent and reverse pathologic cardiac remodeling through the regulation of signaling pathways and molecular mechanisms enumerated below. The experimentally demonstrated actions and potential therapeutic applications of GHRH analogs are summarized in Table 1. These compounds may also counteract oxidative stress and inflammation, affecting favorably the energetics of cardiomyocytes, among other actions on the myocardium (Fig. 2).

**Table 1:**
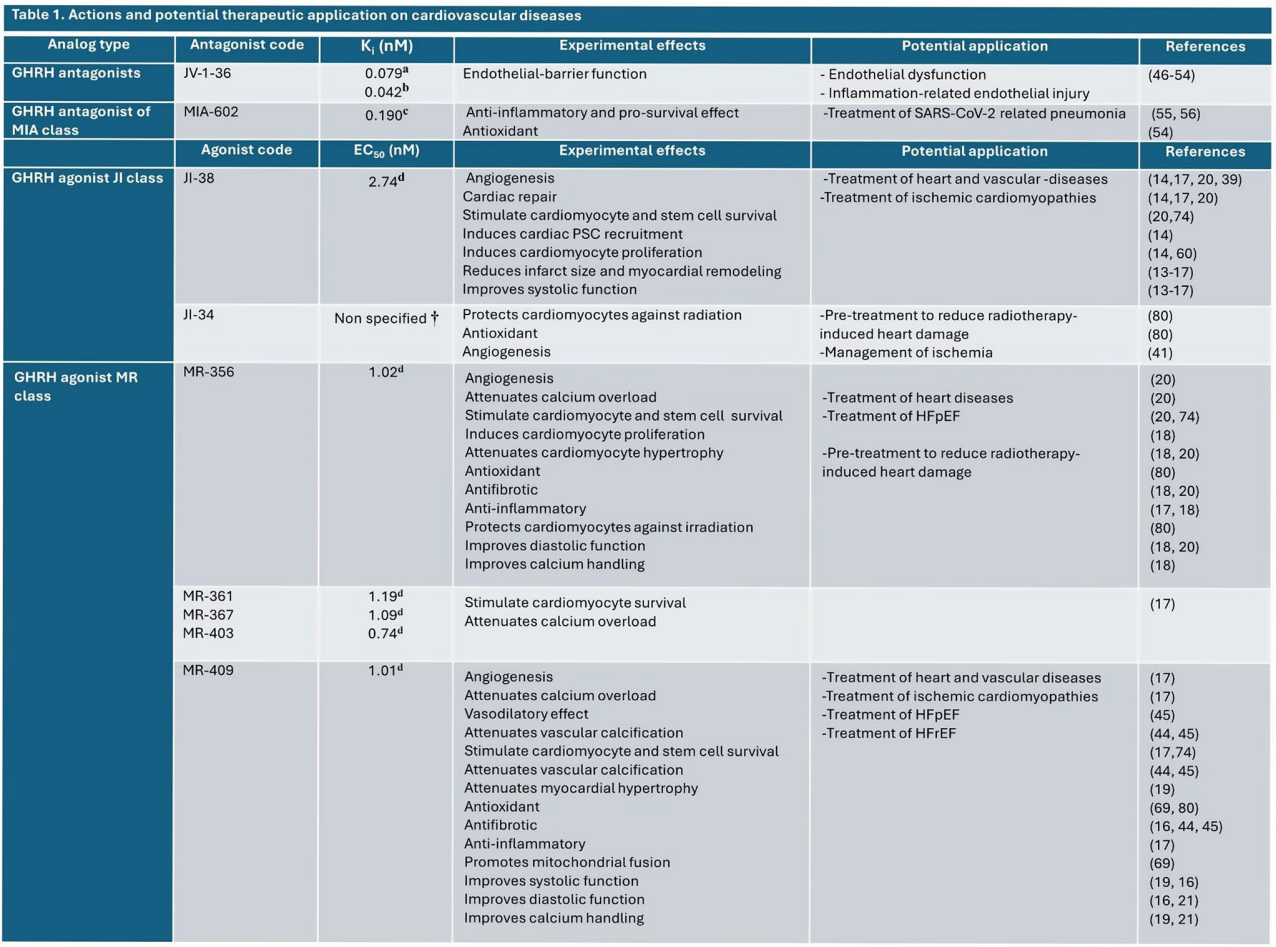
List of GRHR synthetic analogs tested in the cardiovascular system detailing experimentally demonstrated actions and potential therapeutic applications. *K*_*i*_, dissociation constant of the GHRH antagonists. *EC*_*50*_, half maximal effective concentration of the GHRH agonists. ^**A**^ data from *Varga et al.* (2004) [88]; ^**b**^ data from *Varga et al.* (1999) [89]; ^**c**^ data from *Zarandi et al.*. (2017) [12]; ^**d**^ data from *Cai et al.* (2014) [11]; **†** relative affinity of JI-34 compared to standard hGHRH [1–29]-NH2 was reported as 29.35 fold (from *Izdebski et al.* (1995) [90]

### Synthetic GHRH analogs in animal models of cardiac diseases

### 4.1.1 Ischemic cardiomyopathy

**Fig. 3:**
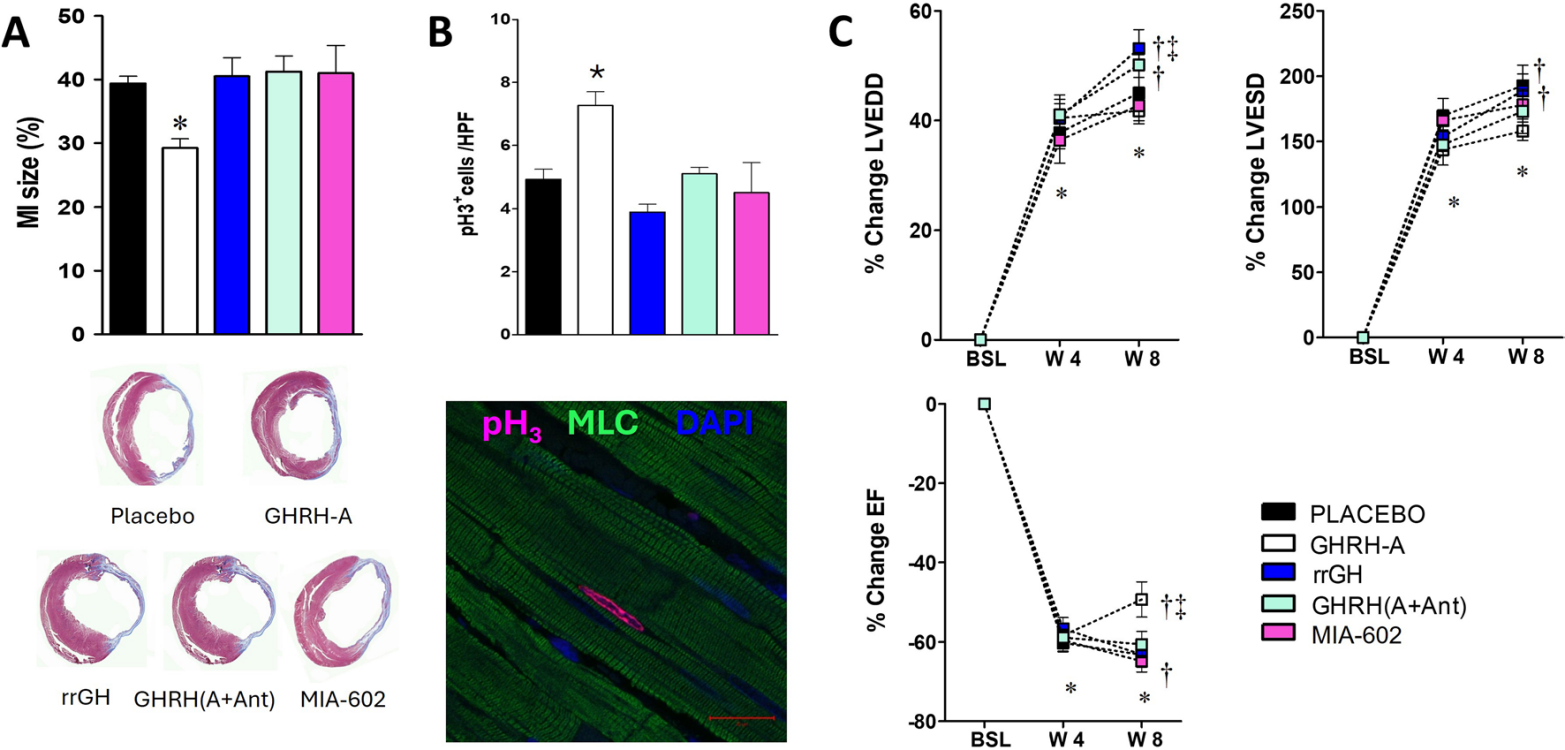
Impact of GHRH agonist administration on infarct size, cardiomyocyte proliferation and ventricular remodeling following myocardial infarction. **A**, Averaged percentage of infarct size (top) and representative images of hearts from every group comparing the extension of the fibrotic scar (bottom). **B**, Averaged abundance of mitotic cardiomyocytes (top) and a representative image of a cardiomyocyte positive for phosphorylated histone H3 (pH3) (bottom). MLC, myosin light chain. **C**, Depicted are changes over time in LV end-diastolic (LVEDD) (Upper Left), end-systolic (LVESD) diameters (Upper Right), and ejection fraction (EF) (Lower). As shown, the GHRH agonist (GHRH-A) offsets the increase in LVESD and LVEDD, resulting in an improvement in EF. The effect of the agonists is blocked by the receptor antagonist MR-602 (Ant) consistent with a receptor mediated effect. Treatment with rrGH does not reproduce the effects of JI-38, All values represent mean ± SEM (*n* = 7–10), *P < 0.05 vs. baseline (BSL), same group; †*P* < 0.05 vs. wk 4 (W4), same group; ‡*P* < 0.05 vs. all other groups at week 8 (W8), except GHRH (A + Ant). §*P* < 0.05 vs. all other groups at W8. Adapted from *Kanashiro-Takeuchi RM et al. 2012*,* PNAS* [14].

The experimental treatments with GHRH agonists on animal models of ischemic cardiac injury have been consistently beneficial [11, 13–17] in comparison with previous studies using GH or IGF-I, which showed inconsistent outcomes [14, 15, 63–67]. In a study by Granata et al. using hearts from rats subjected to ischemia-reperfusion, ex vivo infusion with ratGHRH improved recovery of left ventricular (LV) end-diastolic pressure, LV developed pressure (LVDP) and maximum rise rate of LVDP (dP/dt max) [13]. These positive effects of GHRH on myocardial function were associated with reduced infarct size and increased cardiomyocyte survival, mediated by activation of cardiac GHRHRs and downstream PI3K/Akt signaling pathways. Parallel in vitro experiments on cardiomyoblasts demonstrated the involvement of pro-survival and reparative signaling pathways, including ERK1/2, PI3K/Akt, adenyl cyclase (AC)/cAMP/PKA and phosphorylation of the transcription factor cAMP response element binding protein (CREB). These experiments also suggest that an autocrine/paracrine mechanism involving GHRH secretion plays a role within the myocardium since both treatment with GHRHR antagonist and GHRH gene silencing in myocytes reduced cell survival [13]. Synthetic GHRH agonists containing different amino acid (non-natural) substitutions, were used in vivo to treat infarcted rats [11]. Significant recovery in ejection fraction (EF) was seen with most of the tested compounds; however, only JI-38, MR-356 and MR-409 exhibited reparative capacity as determined by reduction of the infarct size (Fig. 3) [11, 17]. JI-38 not only prevented [15], but also reversed [14] structural (chamber dimensions and fibrosis) and functional (stroke volume, cardiac output, end-diastolic pressure, etc.) cardiac remodeling after myocardial infarction. These studies also demonstrated that the GHRH agonist stimulated the recruitment of cardiac progenitor cells (potentially originating from the neural crest [68]), induced cell proliferation (Fig. 3), and increased capillary density in the myocardium, further contributing to improved cardiac function. Importantly, JI-38 increased the expression of the transcription factor GATA-4 in vivo [14]. GATA-4 plays important roles in regulating cell differentiation, proliferation, organ morphogenesis, and regulation of apoptosis and is also thought to act in adaptive responses as a key regulator of hypertrophy-associated genes in the heart. Rat recombinant GH (rrGH) was used in these studies but showed no functional benefits compared to JI-38 (Fig. 3). In a later study of myocardial infarction in rats [17], MR-409 reduced plasma levels of interleukin (IL)-2, IL-6, IL-10 and tumor necrosis factor-alpha (TNF-α), cytokines related to inflammation and fibrosis. Although the expression of extracellular matrix (ECM)-related genes was up-regulated in MR-409-treated animals, favorable outcomes resulted from the inhibition of pro-apoptotic molecules and pro-fibrotic systems, and by elevation of bone morphogenetic proteins, as shown by in vitro experiments with H9c2 cardiomyoblasts. MR-409 limited the expression of Cdkn1A (or p21) [17, 69], an age-related gene mainly controlled by p53, that can modulate cell cycle, differentiation, apoptosis, senescence, DNA repair [70, 71] and genes related to cardiac remodeling-related molecules [17, 72].

**Fig. 4:**
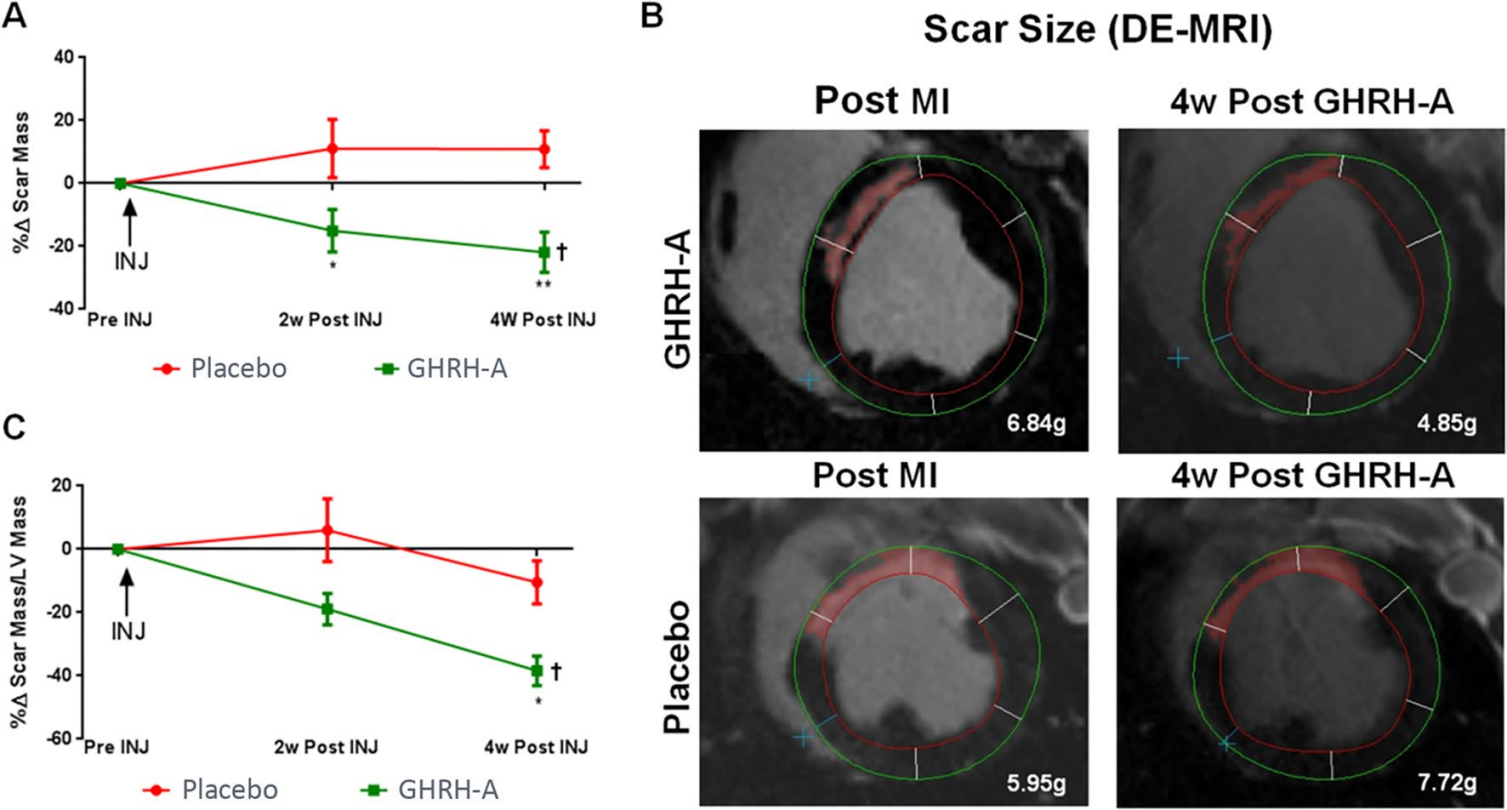
GHRH agonist treatment reduces myocardial infarct size in swine following myocardial infarction. **A**, Percent change of scar mass in the GHRH agonist (GHRH-A) versus placebo group at 2- and 4-weeks (w) post initiation of treatment (2-way ANOVA, between group: *P = 0.04 and **P = 0.003 vs. placebo, respectively). One-way ANOVA: †*P* = 0.02 for GHRH agonist group. **B**, Cardiac Magnetic Resonance images of hearts from an animal in each group before and 4 weeks after the initiation of treatment. **C**, Percent change of scar as a percentage of left ventricular mass in GHRH agonist group versus placebo group at 2- and 4-weeks post initiation of treatment (2-way ANOVA, between group: P = NS and *P = 0.02 vs. placebo, respectively). One-way ANOVA: †*P* = 0.0002 for GHRH agonist group. From *Bagno LL et al. 2015*,* J Am Heart Assoc.* [16]

In a swine model of myocardial infarction, MR-409 treatment reduced scar size (Fig. 4) and improved diastolic function [16]. The expression of GHRHR was increased, but only in the border zone of the infarcted area in GHRH agonist-treated swine compared with the placebo-treated group. Unlike in rats, these effects were not accompanied by improved systolic cardiac function, microvascular density, proliferation or apoptosis markers. However, it is important to emphasize the differences in cardiac remodeling post-myocardial infarction between rodent and swine models. While remodeling can occur rapidly in rodents, the process in swine, similar to human physiology, may require a longer follow-up to identify changes after treatment [73]. These differences need to be carefully addressed before a definitive conclusion can be made.

### 4.1.2 Non-ischemic cardiomyopathies and heart failure with preserved ejection fraction (HFpEF)

**Fig. 5:**
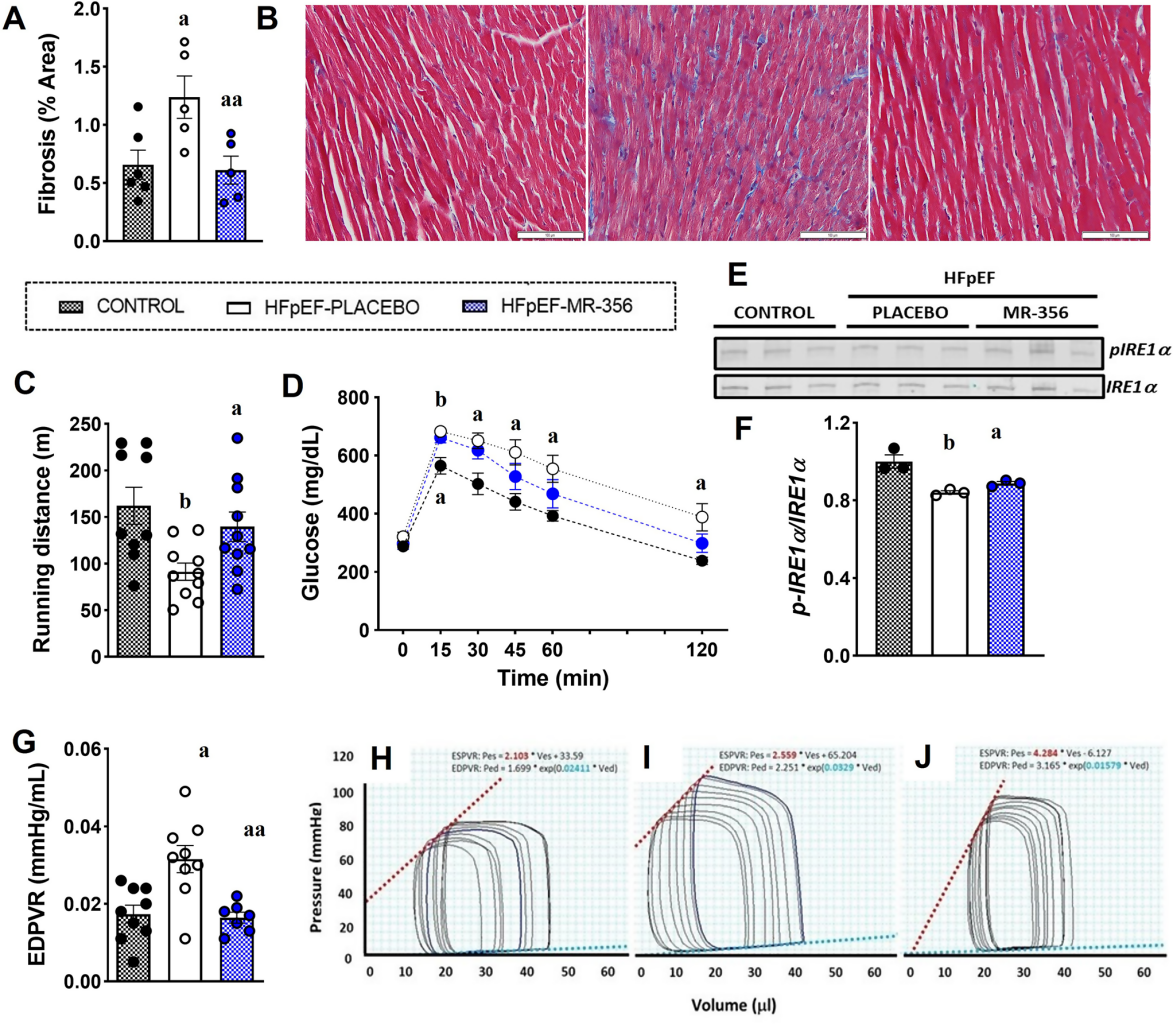
Cardiac performance and hemodynamic changes in cardiometabolic HFpEF induced by 9 weeks of HFD + L-NAME. **A**, Quantification of interstitial fibrosis in hearts from mice: control, HFpEF treated with placebo (HFpEF-placebo) and HFpEF treated with MR-356 (HFpEF-MR-356). One-way ANOVA followed by Tukey’s test, ^**a**^*P* < 0.05 vs. control, ^**aa**^*P* < 0.05 vs. HFpEF-placebo, *n* = 4 or 6. **B**, Representative images of Masson-Trichrome staining from each group. **C**, Exercise tolerance test showed a reduced running capacity in the HFpEF-placebo mice compared with control and HFpEF-MR-356 mice (^**b**^*P* < 0.01 vs. control, ^**aa**^*P* < 0.05 vs. HFpEF-placebo by unpaired t test, *n* = 9 or 10). **D**, Intraperitoneal glucose tolerance test (ipGTT) showed increased glucose levels in HFpEF-placebo mice compared with control mice in all time points after glucose injection, whereas MR-356-treated mice showed an increase in glucose levels only at 15- and 30-min time point, two-way ANOVA followed by Sidak multiple-comparisons test, ^**a**^*P* < 0.05, ^**b**^*P* < 0.01 vs. control, *n* = 9. **E**, Representative immunoblots of phosphorylated IRE1a (top) at Ser724 and total IRE1a (bottom). **F**, Western blot quantification showing reduced ratio of phospho-IRE1a to IRE1a in HFpEF-placebo mice compared with control (one-way ANOVA followed by Tukey’s test, ^**a**^*P* < 0.05 vs. control, ^**aa**^*P* < 0.05 vs. HFpEF-placebo, *n* = 6), **G**, Slope of end-diastolic pressure-volume relationship (EDPVR; E),: Kruskal–Wallis test followed by Dunn’s multiple-comparisons test, ^**a**^*P* < 0.05, vs. control, ^**aa**^*P* < 0.05 vs. HFpEF-placebo, *n* = 9 or 10.**H–J**, Representative pressure-volume (P-V) loops from control (H). Adapted from *Kanashiro-Takeuchi RM et al. 2023*,* Am J Physiol Heart & Circ Physiol.* [20]

A study by Gesmundo et al.. using a murine model of transverse aortic constriction (TAC)-induced pressure overload [19], which exhibits characteristics of HF with reduced EF (HFrEF), demonstrates that MR-409 attenuated gene expression of several cardiac markers of hypertrophy, including myosin heavy chain 7 (MYH7), natriuretic peptides (NPPA and NPPB) and a-actin (Acta1), and improved myocardial (in vivo) and cardiomyocyte (in vitro) function. This study demonstrated that pathological pathways activated by β-adrenoreceptor stimulation with phenylephrine (PE) and mediated by the exchange protein directly activated by cAMP 1 (Epac1), are efficiently blocked by the activation of a parallel cAMP/PKA signaling pathway triggered by the GHRH agonist. In recent studies in murine models of HF with preserved ejection fraction (HFpEF), either in mice subjected to low dose Angiotensin II (Ang-II) infusion [18] or mice fed a diet of high-fat chow plus the NOS inhibitor N(ω)-nitro-L-arginine methyl ester (L-NAME) in the drinking water [20], ex vivo analysis of cardiomyocyte size and cross-sectional area in heart sections showed that MR-356 abolished the cardiomyocyte hypertrophy. However, in vivo, treatment with MR-356 did not prevent or reverse ventricular hypertrophy, in contrast to the observations in the TAC-induced pressure overload model [19]. The absence of a significant reduction in heart weight and ventricular mass has been attributed to induction of proliferation in vivo [18, 74], consistent with the findings in the models of ischemic cardiomyopathy described above [14, 17]. These effects were accompanied by an anti-fibrotic effect in both murine models of HFpEF. Reduction of cardiac fibrosis is a characteristic effect of GHRH agonists independent of the myocardial injury model. Although the specific molecular mechanism(s) underlying the antifibrotic effect remains unclear, the anti-inflammatory properties of GHRH and GHRH agonists are well documented [17, 18, 20, 21]. Accordingly, in models of HFpEF, GHRH agonist treatment abolished pro-inflammatory and pro-fibrotic signaling factors and mediators such as transforming growth factor-beta (TGF-β), monocyte chemotactic peptide-1 (MCP-1), α-smooth muscle actin (α-SMA), vascular endothelial growth factor (VEGF-A), and NOS2 in the myocardium. These effects might suggest that activation of myocardial GHRHRs by these agonists inhibits pro-inflammatory signaling pathways, as demonstrated in other tissues [62], preventing the pathologic deposition of collagen in the extracellular matrix (Fig. 5). In vitro studies indicate that a significant part of the effects enumerated above are induced by direct activation of GHRHRs on cardiac fibroblasts, as the tumor necrosis factor-alpha (TNF-α) gene expression induced by 1 nmol/L Ang-II in cultured cardiac fibroblasts was partially reverted by treatment with MR-356 [18].

Overall, administering MR-356 to mice with HFpEF improved myocardial performance associated with the restoration of diastolic function (Fig. 5). In the HFD + L-NAME (cardiometabolic HFpEF) murine model, this GHRH agonist restored exercise capacity, ameliorated glucose intolerance and mitigated endoplasmic reticulum (ER) stress by targeting the molecular mechanisms of the UPR (Fig. 5) [20]. While MR-356 prevented the increase in aortic blood pressure induced by Ang-II in the 4 weeks experimental model (when the treatment was initiated simultaneously with the Ang-II-infusion), Kanashiro-Takeuchi et al. did not detect a reduction in arterial hypertension in mice with an established cardiometabolic HFpEF phenotype [20]. The administration of L-NAME prevents vasodilation [75, 76]. Therefore, the effect of GHRH agonist on blood pressure in this model may be diminished, and although activation of the sGC/cGMP pathway was detected in isolated cardiomyocytes, further specific studies aimed to determine whether this GHRH agonist has a blood pressure-lowering effect per se on the peripheral vasculature are needed.

Direct activation of GHRHRs on isolated murine cardiomyocytes with murine recombinant GHRH induced increasing intracellular levels of cAMP and cGMP independently of the involvement of GH or IGF-I [13, 18, 19]. However, in vivo, the participation of GH and/or IGF-I cannot be ruled out as mediators of the overall beneficial effects of GHRH and GHRH agonists.

In clinical settings, characteristics compatible with the HFpEF syndrome are commonly observed in older individuals. A relevant model system is aged mice, where treatment with MR-409 improved cardiomyocyte energetics (see below), mitochondrial morphology, and systolic and diastolic function [69]. In this model of myocardial dysfunction, MR-409 restored the immune system profile and decreased the macrophage abundance in the heart, thus modulating the inflammatory environment associated with aging [77]. Collectively, these effects mitigate the cardiovascular burden associated with aging and increase the survival of old mice.

In a novel porcine cardiorenal model of HFpEF induced by chronic kidney disease (12 weeks post renal artery embolization) [21], Rieger and colleagues showed that treatment with MR-409 for 4–6 weeks improved several aspects of diastolic hemodynamics and myocardial function, including normalization of the LV end-diastolic pressure, reduction of LV end-diastolic pressure-volume relationship and amelioration of the stroke work. The treatment with this agonist also reduced the myocardial levels of pro-BNP and increased the Ca^2+^ transient amplitude versus placebo-treated animals. These results have important implications because of the high prevalence of chronic kidney disease-induced HFpEF and the relevance of testing these GHRH agonists in a large animal model that resembles the pathologic phenotype observed in humans.

## GHRH effects on myocardial energetic and redox balance

Reactive oxygen species (ROS) and reactive nitrogen species (RNS) represent very important components of the intracellular signaling pathways, which are tightly regulated by redox-sensitive mechanisms. Dysregulation of this equilibrium leads to nitroso-redox imbalance and subsequent oxidative/nitrosative stress, affecting myocardial function [78, 79]. GHRH analogs exhibit redox properties, and much evidence suggests that these properties involve activation of GHRHR downstream signaling and activation of antioxidant mechanisms targeting ROS rather than merely resulting from a chronic alleviation of the pathologic phenotype. Paradoxically, the ability of GHRH analogs to antagonize ROS has been demonstrated in both GHRH agonists and antagonists. Accordingly, Shen and colleagues demonstrated, in smooth muscle cells, that MR-409 inhibits nicotinamide adenine dinucleotide phosphate (NADPH) oxidase activity and ROS generation by a cAMP/PKA-mediated de-activation of NADPH oxidase [44]. Moreover, oxidative stress in cardiomyocytes induced by either doxorubicin [69] or radiation [80] was attenuated by treatment with MR-409 or JI-34 and MR-356, respectively. In neonatal cardiomyocytes, irradiation with high-energy photon beams mediated the ROS-induced protein kinases activation (ERK1/2 and Akt) which was counteracted by JI-34 or MR-356 [80]. This contradictory effect of GHRH agonists inhibiting ERK1/2 and Akt activities might be explained as consequence of ROS scavenging [44] rather than a direct GHRHR-mediated inhibition of the kinase activation pathways (which would be inconsistent with previous findings [13, 74]). Under non-stress conditions, iNOS is minimally expressed in normal hearts; however, stress induces iNOS expression in cardiomyocytes and generates oxidative/nitrosative stress and mitochondrial dysfunction [81]. Kanashiro-Takeuchi et al. showed that MR-356 treatment reduces the elevated protein levels of iNOS in the murine model of cardiometabolic HFpEF [20], ameliorating the oxidative milieu. In the vascular system, the story appears to be different. In fact, GHRH can induce ROS generation possibly through activation of the JAK2/STAT3 pathway [60], which is abolished by the GHRH antagonist Acetyl-(D-Arg2)-GRF (1-29) amide [36]. Furthermore, the antagonist MIA-602 protected microvascular endothelium integrity from hydrogen peroxide-induced damage [54]. Surprisingly, Recinella et al. showed that in brain, both GHRH agonist (MR-409) and GHRH antagonist (MIA-690) induce the nuclear factor erythroid 2-related factor 2 (Nrf2) and target genes, which mediate the intracellular antioxidant response [62]. In this particular study, the GHRH analogs reduced the inflammatory profile after a lipopolysaccharide (LPS) insult. Nrf2-regulated enzymes are an essential component of the pathogenesis of cardiovascular diseases [82] and are strongly associated with HF. Compelling evidence in multiple organs demonstrates that either GHRH antagonists or GHRH/GHRHR signaling disruption (ablation) [25, 83, 84] activate the Nrf2-mediated antioxidant response and contribute to the anti-inflammatory effects, also associated with longevity in murine models [25, 85]. Thus, the role of GHRHR signaling on antioxidant responses might strongly depend on the specific pathologic environment, tissue/cellular type and signaling pathways favored by the GHRHR splice variant.

The study from Xiang et al.. in aged mice showed that MR-409 treatment promoted mitochondria fusion [69]. This process was shown to be a PKA-mediated regulation of the posttranslational metabolism of Opa-1 (a protein that participates in the fusion of mitochondria). Parkin, a mitophagy-related protein was reduced by MR-409, thus protecting integrity and preventing fragmentation of mitochondria. Treatment with MR-409 also improved oxygen consumption rate (OCR) and up-regulated pathways of oxidative phosphorylation, which indicates enhanced electron transport chain and, therefore, mitochondrial function. In contrast, in human induced pluripotent stem cell (hiPSC)-derived cardiomyocytes, recombinant human GHRH treatment induced metabolic changes associated with HIF-1a targeted genes leading to enhanced glycolysis and augmented ATP synthesis, without changes in OCR [33]. These results suggest that, in developing cardiomyocytes, GHRHR activation promotes adjustments to the energetic metabolism, making hiPSC-cardiomyocytes reliant on glycolysis rather than oxidative phosphorylation.

## GHRHR signaling targets calcium handling and sarcomere proteins

**Fig. 6:**
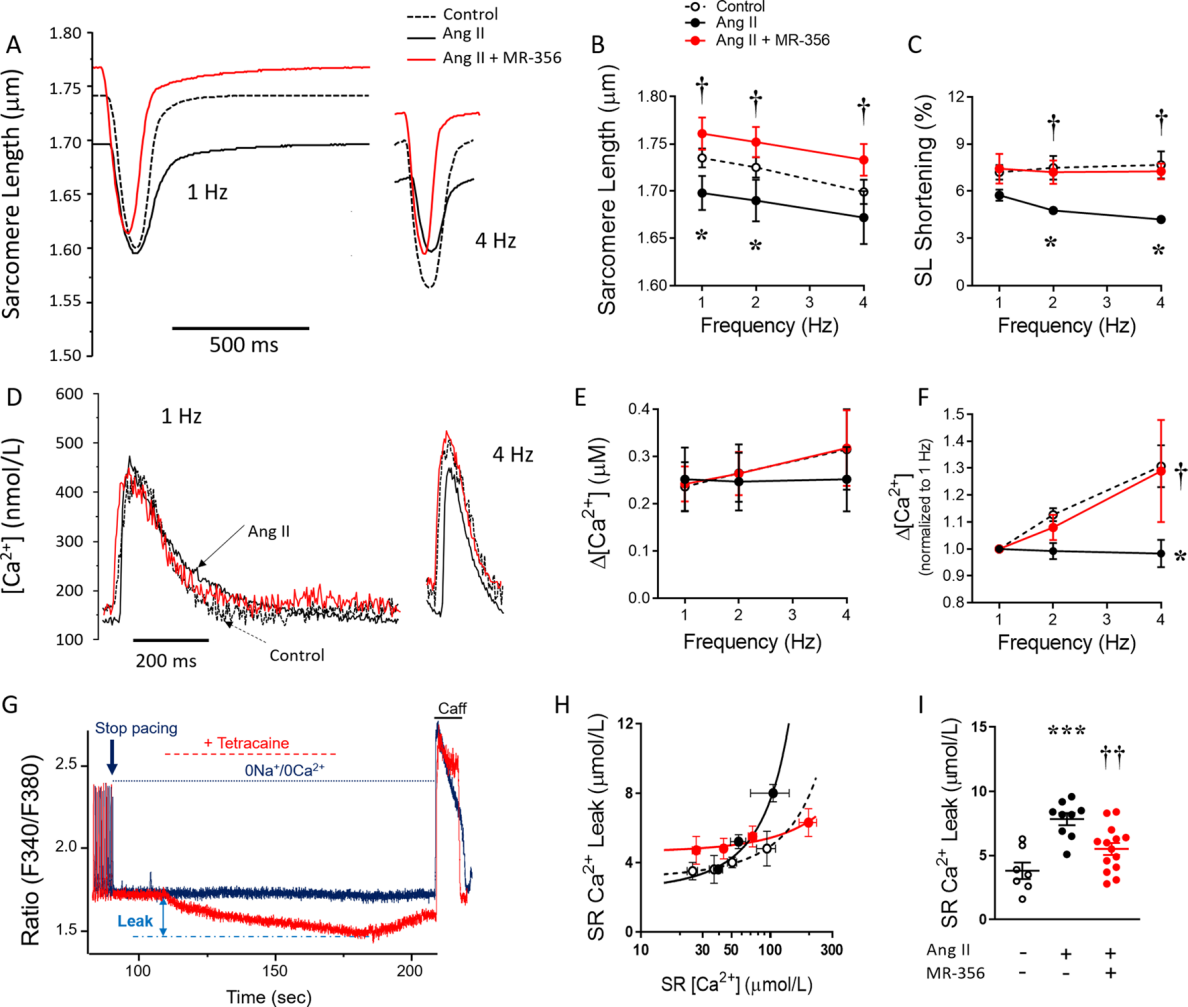
Sarcomere dysfunction, depressed contractile reserve and calcium handling alterations in the Ang-II-induced model are prevented by co-treatment with the GHRH agonist MR-356. **A**, Representative traces of sarcomere twitches of cardiomyocytes, electric field stimulated at 1- and 4 Hz, from male CD1 control mice (*N* = 4, *n* = 30 cells) and mice that underwent either Ang-II (*N* = 5, *n* = 41 cells) or Ang-II + MR-356 treatment (*N* = 5, *n* = 38 cells). **B**, Resting sarcomere length (SL) in cardiomyocytes from each group at different pacing rates. **C**, Sarcomere shortening in response to pacing. (***D***) Representative traces of intracellular [Ca^2+^] in electric field-stimulated cardiomyocytes. (***E***) Δ[Ca^2+^] transient amplitude in response to pacing. **F**, Δ[Ca^2+^] normalized (with respect to 1 Hz) in response to pacing. **G**, Representative traces of intracellular [Ca^2+^] in cardiomyocytes illustrating the method for measuring SRCa^2+^ leak using tetracaine. Upper (Blue) trace corresponds to Ca^2+^ from a cardiomyocyte not exposed to tetracaine; lower (red) trace is the Ca^2+^ from the same cardiomyocyte treated with tetracaine for 70 s after pacing was stopped. **H**, SRCa^2+^ leak-load relationship in cardiomyocytes from control (*N* = 4 mice, *n* = 31 cells), Ang-II-treated (*N* = 5 mice, *n* = 38 cells) or Ang-II + MR-356-treated (*N* = 5 mice, *n* = 35 cells) CD1 mice. **I**, Average and individual SRCa^2+^ leak in cardiomyocytes from all groups at matched SRCa^2+^ load = 99.1 µmoL/L. From each mouse, a total of 5–9 cells were studied. *P < 0.05, ***P < 0.001 in Ang-II vs. control, and †*P* < 0.05, ††*P* < 0.01 in Ang-II + MR-356-treated vs. Ang-II; one- or two-way ANOVA as appropriate. From *Dulce RA et al. 2022*,* Cardiovasc Res* [18].

Most of the molecular mechanisms and signaling pathways addressed above, including transcription factors, protein kinases, ROS signaling and metabolic performance, impact the machinery that underlies the contractile function of the myocardium, i.e., the processes that mediate the transduction between the depolarization and the contraction of the myocardium, are together known as “excitation-contraction coupling”. Alterations in protein expression and posttranslational modifications (such as phosphorylation among many others) of Ca^2+^ handling- and sarcomere-related proteins directly affect the contractile performance of the heart. Across different experimental models of cardiovascular pathologies, GHRH analogs have favorable effects on these aspects. Thus, cardiomyocytes isolated from the TAC-induced pressure overload mice chronically treated with MR-409 exhibit improved cell shortening and relaxation compared with those from placebo-treated mice [19]. These effects were preceded by a faster rising phase of the Ca^2+^ transient, an improvement associated with restoration of the crest morphology of the sarcolemma (better T-tubule organization). Curiously, although MR-409 restored the expression of the sarcoplasmic reticulum (SR) Ca^2+^-ATPase 2a (SERCA2a), a protein responsible for the Ca^2+^ re-uptake into the SR (intracellular Ca^2+^ stores), a delay in the Ca^2+^ decay rate was observed. This result might appear contradictory; however, the authors showed that in cardiomyocytes stimulated with phenylephrine, the GHRH agonist reduced the phosphorylation of phospholamban (PLB) at threonine 17. PLB is a small regulatory protein that in its dephosphorylated state binds to SERCA2a slowing the Ca^2+^ uptake rate. PLB phosphorylation induces detachment and therefore faster functioning of SERCA2a. In this regard, cardiomyocytes isolated from mice subjected to Ang-II-induced HFpEF, which exhibited depressed response to increasing frequency of stimulation in terms of sarcomere shortening and Ca^2+^ cycling performance, showed improved response when treated with MR-356 (Fig. 6) [18]. Faster sarcomere relaxation and Ca^2+^ decay rate were associated with higher PLB phosphorylation (at serine 16 and threonine 17), despite the downregulation of SERCA2a expression induced by Ang-II not being prevented. Abnormal SR Ca^2+^ leak, which is increased in this model, likely mediated by dysfunctional Ca^2+^ gating of the SR channel ryanodine receptor 2 (RyR2), was reduced by treatment with MR-356 (Fig. 6) [18]. The elevated Ca^2+^ leak impacts the energy demands necessary to compensate for the extra flux of Ca^2+^ into the cytosol, thus contributing to the depressed inotropic response in HF. With respect to the contractile apparatus, myofibrillar proteins play an important role in responding to the Ca^2+^ transient and thus transducing the signal into a mechanical effect (contraction). Proteins of the troponin complex respond to Ca^2+^ allowing the interaction of thin and thick filaments and exerting a regulatory function. Myosin binding protein C (MyBPC) on the thick filaments also modulates sarcomere contraction/relaxation. Phosphorylation of cardiac troponin I (cTnI) and cardiac MyBPC is increased by MR-356 treatment in murine models of HFpEF [18, 20], favoring the dynamic interaction of myofilaments with a consequent improvement in myocardial relaxation. Phosphorylation of the giant sarcomeric protein Titin is restored by treatment with GHRH agonists in mice (MR-356) [20] and swine (MR-409) [21] models of HFpEF. Impaired titin phosphorylation is associated with stiffness and diastolic dysfunction.

In summary, activation of GHRHR prevents and reverses dysfunctional cardiomyocyte performance by targeting myofibrillar and Ca^2+^ handling proteins, thus enhancing both inotropic and lusitropic functions.

## Pharmacologic perspectives

Among a spectrum of GH secretagogues, many synthetic GHRH agonists have been developed, but few have been tested in experimental heart diseases [11, 13–16, 18–21]. Among the many potential candidates, some promote myocardial repair, although they differ in binding affinity to the GHRHRs or potency to induce GH release [11]. Therefore, it is reasonable to expect that these subtle but distinctive characteristics might determine that a given compound preferentially favors one or another cardiac repair pathway, e.g., improving diastolic function over systolic function or vice versa (Table 1). Future studies are needed to optimize and pharmacologically test GHRH analogs for indication-specific translational development.

While these compounds offer numerous benefits and have shown promising results, their administration presents challenges to widespread application. Given their polypeptide nature [86], GHRH synthetic analogs are susceptible to degradation in the digestive tract. Although the synthetic derivatives are significantly more stable than GHRH [11, 12], they exemplify the challenge of selecting a route of administration that bypasses this process. Thus, injectable versions of these compounds should be administered intravenously or subcutaneously. To maintain consistent blood levels of these polypeptides, which are rapidly metabolized and cleared from the bloodstream within a few minutes [11], daily injections are necessary. Therefore, a subcutaneous route, although minimally invasive, might be preferred. Novel delivery methods are being developed to improve stability and sustained release of GHRH analogs, such as the use of BSA/heparin nanoparticles loaded with the analog to target specific affected sites [87]. Further investigations to optimize these therapeutic approaches are needed.

## Conclusion

In summary, GHRHRs are widely present throughout the cardiovascular system, indicating that GHRH can act directly on these tissues largely independently of the GH/IGF-I axis. Much experimental data supports that GHRH and its analogs may counteract maladaptive pathologic cardiovascular conditions at different functional levels in a broad spectrum of cardiovascular diseases. In addition to the activation of signaling pathways that regulate inotropy and lusitropy, these synthetic compounds positively affect myocardial energetics, which directly impacts the excitation-contraction coupling efficiency, thus contributing to the improvement in myocardial performance. Moreover, they can ameliorate harmful responses such as oxidative stress and inflammation, and reverse pathologic remodeling including cardiac hypertrophy and fibrosis. There is a paucity of mechanistic understanding regarding the vasoactive capacity of GHRHR activation, but studies suggest that synthetic antagonists of GHRH play a protective role in endothelial integrity and function. Based on the compelling evidence for the role of GHRHR signaling in cardiac repair, GHRH/GHRH analogs are promising therapeutic candidates for multiple cardiomyopathies (Table 1) and further clinical translational research is needed.

## Data Availability

No datasets were generated or analysed during the current study.

## Abbreviations

AC: Adenyl Cyclase
Akt: Ak Strain Transforming or Protein Kinase B
Ang-II: Angiotensin II
ATP: Adenosine Triphosphate
cAMP: cyclic Adenosine Monophosphate
Cdkn1A: Cyclin-dependent kinase inhibitor 1 A
cGMP: cyclic Guanosine Monophosphate
cMyBPC: cardiac Myosin-Binding Protein C
cTnI: cardiac Troponin I
ECM: Extracellular Matrix
EF: Ejection Fraction
eNOS: endothelial Nitric Oxide Synthase
ER: endoplasmic Reticulum
ERK1/2: Extracellular Signal-Regulated Kinases ½
GH: Growth Hormone
GHRH: Growth Hormone-Releasing Hormone
GHRHR: GHRH Receptor
HF: Heart Failure
HFD: High Fat Diet
HFpEF: HF with preserved EF
HFrEF: HF with reduced EF
HIF-1α: Hypoxia-Inducible Factor-1 alpha
hiPSC: human induced Pluripotent Stem Cell
IGF-I: Insulin-like Growth Factor-I
IGHD: Isolated Growth Hormone Deficiency Type I
IL: Interleukin
iNOS: inducible Nitric Oxide Synthase
L-NAME: N(ω)-Nitro-L-Arginine Methyl Ester
LPS: Lipopolysaccharide
LV: Left Ventricle
LVDP: LV Developed Pressure
MCP-1: Monocyte Chemotactic Peptide-1
NADPH: Nicotinamide Adenine Dinucleotide Phosphate
NO: Nitric Oxide
Nrf2: Nuclear factor erythroid 2-related factor 2
OCR: Oxygen Consumption Rate
Opa-1: Optic atrophy 1
PE: Phenylephrine
PI3K: Phosphatidyl Inositol 3 Kinase
PKA: Protein Kinase A
PKC: Protein Kinase C
PLB: Phospholamban
RNS: Reactive Nitrogen Species
ROS: Reactive Oxygen Species
RyR2: Ryanodine Receptor 2
SERCA2a: Sarcoplasmic/Endoplasmic Reticulum Ca2+-ATPase 2a
sGC: soluble Guanylyl Cyclase
SMA: α-Smooth Muscle Actin
SR: Sarcoplasmic Reticulum
TAC: Transverse Aortic Constriction
TNF-β: Transforming Growth Factor-Beta
TH: Tyrosine Hydroxylase
TGF-α: Tumor Necrosis Factor-Alpha
UPR: Unfolded Protein Response
VEGF-A: Vascular Endothelial Growth Factor
